# Special Grain Boundaries in Ultrafine-Grained Tungsten

**DOI:** 10.1186/s11671-016-1488-4

**Published:** 2016-07-15

**Authors:** O. V. Dudka, V. A. Ksenofontov, E. V. Sadanov, I. V. Starchenko, T. I. Mazilova, I. M. Mikhailovskij

**Affiliations:** Department of Condensed Matter, National Science Center “Kharkov Institute of Physics and Technology”, Nat. Acad. of Sci. of Ukraine, Academicheskaja Str. 1, Kharkov, 61108 Ukraine

**Keywords:** Nanostructure, Grain boundaries, Field ion microscope, Tungsten

## Abstract

Field ion microscopy and computer simulation were used for the study of an atomic structure high-angle grain boundary in hard-drawn ultrafine-grained tungsten wire. These boundaries with special misorientations are beyond the scope of the coincident site lattice model. It was demonstrated that the special non-coincident grain boundaries are the plane-matching boundaries, and rigid-body displacements of adjacent nanograins are normal to the <110> misorientation axis. The vectors of rigid-body translations of grains are described by broad asymmetric statistical distribution. Mathematical modeling showed that special incommensurate boundaries with one grain oriented along the {211} plane have comparatively high cohesive energies. The grain-boundary dislocations ½<110> were revealed and studied at the line of local mismatch of {110} atomic planes of adjacent grains.

## Background

The huge advances in experimental characterization methods and mathematical modeling at the nanoscales have catalyzed the appearance of a new era in the area of structural materials. Studies of grain boundaries (GBs) in ultra-strength nanomaterials are of immense scientific interest. Grain boundaries have significant effects on the mechanical properties of a wide range of polycrystalline structural materials [1–3]. The role of GBs in these ultrafine-grained materials is enhanced due to the extremely increased amount of GBs, their total area, and the corresponding fraction of near-boundary atoms. The study of atomic-level structure of GBs has been greatly encouraged by the use of high-resolution transmission electron microscopy from which detailed information about the grain-boundary microtopography, defects, and local atomic arrangement can be obtained. It was recognized that the properties of nanocrystalline materials are determined to a large extent by the properties of their GBs. Since the physical and mechanical properties of GBs are inevitably dependent on their atomic structure, the study of the GB structure has emerged as an essential area in the larger field of the science and design of ultrafine-grained materials.

In spite of the progress of other microscopic techniques, field ion microscopy is still the only means by which the individual atoms on irregular GBs of general type can be resolved. The perspective of the field ion microscope (FIM) as a metallurgical tool was clearly demonstrated by Müller [4] in the fifties. In the early sixties, field ion microscopy was able to prove [5] that the crystal lattice disturbances in the boundary area are restricted to a region no greater than one or two atom spacings. As a result, the conventional model of an amorphous boundary layer could thus be finally discarded. In the issue of early FIM studies [6], the coincidence site lattice (CSL) model of high-angle grain boundaries was developed. However, these early and more recent studies have usually been confined to coherent CSL boundaries with a low reciprocal density of coincident sites. Mathematical simulations of a truly general GB are limited by the use of periodic boundary conditions in the simulation. The majority of the GBs in nanocrystalline tungsten is of general character; just they mainly determine the fundamental properties of this material. Despite their preponderance, the class of general grain boundaries is the least studied and understood.

In our recent study, it was shown that the misorientation angle distribution of GBs in heavily drawn ultrafine-grained tungsten demonstrates a tendency for high-angle GBs to have significant populations at certain non-CSL misorientation angles around the [110] axis [7]. A crystallographic consideration shows that these misorientations are related to a special type of GBs terminated by a low index plane and the medium index complements to this surface. A mathematical modeling showed that the calculated cleavage energy is a function of the misorientation angle for these GBs; incommensurate GBs with special misorientation angles have comparatively high cohesive energies. The GBs have revealed a high resistance to mechanical stresses approaching a significant fraction of the theoretical strength [8]. The goal and scope of the present paper are to investigate the translation states of the special non-CSL tilt GBs in tungsten by using FIM and atomistic computer simulations. Tungsten was chosen as an important plasma facing material for fusion devices with its superior thermo-mechanical features and its extremely low erosion. Due to high melting temperature and extremely small sputtering yield, polycrystalline tungsten is one of the best candidates to withstand the extremely severe radiation conditions in fusion reactors [9].

## Methods

The experiments were carried out in a two-chamber FIM with needle-shaped specimens cooled to 77 K. The microscope chamber was pumped down to the background pressure of 10^−6^ Pa by cryogenic pumps. Field ion images were formed by using helium atoms under a pressure of 10^−4^–10^−3^ Pa. A channel plate image converter [10] was used for FIM image intensification. A continuous set of FIM was recorded by a charge-coupled device camera. The specimens were prepared from 0.15-mm ultrafine-grained tungsten wires of 99.98 at.% (0.01 at.% Si, 0.002 at.% Fe, 0.005 at.% Mo) with a <110> axial texture, which had been manufactured by drawing 10-cm metal-ceramic rods at 700–770 K. The drawn tungsten wire has a nanoscale fiber structure (an average fiber diameter is about 100 nm). Needle-shaped specimens (field emitter tips) with an initial radius of the curvature of about 20 nm at their top were made by electrolytic polishing the wires in 1 N NaOH aqueous solution at alternating voltage of 4–10 V. The specimens had the taper angle near a hemispherical apex in the range of 2–5°. The specimen surface was cleaned and polished in situ in the FIM chamber using controlled field evaporation [10]. The FIM images of the bicrystalline specimen studied in this paper was acquired using the imaging gas under a pressure of 10^−3^ Pa at a positive operating voltage of about 8 kV.

The magnification of FIM images was determined using the value of the radius of curvature *r* by counting the atomic steps between two poles of known angular separation in the FIM micrograph [10]. The crystallographic relationships of adjacent nanograins were extracted from FIM images using standard stereographic methods [10–12]. Miller indices of the crystallographic poles can be assigned precisely once the main symmetry elements of the specimen have been determined [10]. The misorientation angle in a bictystal with known Miller indices was determined by using the usual crystallographic procedures on a stereogram [13]. The bicrystalline field ion specimen studied in detail in this paper can be described as rotations about an axis which, within the experimental uncertainty (±2°), coincides with the center of the projection. In such a case, stereographic constructions are unnecessary and the misorientation angle was measured directly with a protractor. The accuracy on the misorientation angle determination for GBs from the individual FIM images is about ±2°. To increase the accuracy of measurement, the misorientation angles were calculated from a large set of FIM images obtained during controlled field evaporation. Using about a hundred FIM, micrographs provided the accuracy of misorientation angle determination not worse than ±1°. In this case, the calculation of the GB plane orientation was made with an accuracy of about ±2°.

## Results and Discussion

**Fig. 1:**
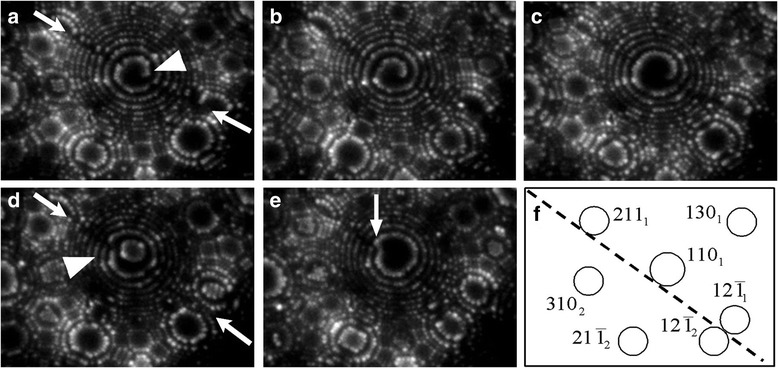
Helium FIM images of the dislocation (shown by *arrow heads*) at the special non-CSL boundary with misorientation angle 19° recorded during an atom-by-atom field evaporation showing the violation of continuity of the {110} circular atomic steps across the boundary before (**a**–**c**) and after (**d**), (**e**) a local GB slip event, and corresponding stereographic projection (**f**). Imaging conditions: field strength of 44 V/nm, *T* = 77 K

The FIM patterns consist of the concentric circular atomic steps of predominant {110}, {111}, and {112} crystallographic planes (Fig. 1). Figure 1 is found to contain a GB with a misorientation angle 19° (shown by arrows) that corresponded to a special non-CSL angle *ω*_*Σ*9_/2 = 19.47*°*, earlier revealed in [7]. The grain boundary is oriented at the average along the $\left(1\overline{1}\overline{2}\right)/\left(1\overline{1}\overline{1}\right)$ boundary plane. FIM images show that, as distinct from the GBs of the general type [9, 10], the special non-CSL boundary is not subjected to preferential field evaporation. It points out that this interface has a relatively low GB energy and correspondingly high cleavage energy.

While the atomic structure and nanotopography of various non-CSL special boundaries are different, there is a clear similarity in the state of rigid-body translation [14, 15] of neighbor grains. The FIM images in Fig. 1 have revealed matching of atomic steps of the {110} planes at the interface. Such a peculiarity of the GB atomic topography points out that the {110} crystallographic planes stay planar through the boundary without rigid translation along the <110> direction. The rigid-body displacement *T* of adjacent grains at the GB in the direction *n* along the <110> axis of misorientation is absent (*Tn*_110_ = 0), in agreement with mirror symmetry of body centered cubic crystals with respect to the {110} crystallographic plane. So, special non-CSL GBs have no lateral component of rigid-body shift along the [110] axis. A similar behavior is characteristic of studied earlier plane-matching GBs in metals [3].

An anti-clockwise spiral can be seen in the middle of the [110] pole in the FIM image (Fig. 1a) representing the region of emergence of the ½<110> GB dislocation at the surface. The GB dislocation is revealed at the line of local mismatch of {110} atomic planes of adjacent grains (shown by an arrow head). The disregistry of these planes is not detectible beyond the dislocation core. The spiral in Fig. 1 shows the contrast from a perfect GB dislocation with the core lying wholly inside the first imaged ring. The circular atomic steps are broken only near the region of emergence of the GB dislocation at the surface. As is shown in [13], such an observation leads to the conclusion that the width of the dislocation core is equal or less than the width of the ledge in which the dislocation emerged calculated from the tip radius. Analysis of more than 10^3^ FIM micrographs showed that the half width (or radius) of the dislocation core is less than or equal to 6 ± 2 Å.

A perfect GB dislocation with the Burgers vector *b* and line vector *l* converts a set of parallel crystallographic planes into helicoids with spirals steps, providing that *nb* ≠ 0 and *nl* ≠ 0. This observation of the ½<110> GB dislocation indicates that special non-CSL GB structures are relaxed so as to survive the {110} close-packed atomic layers in the GB core. During field evaporation, the GB dislocation was abruptly shifted along the boundary to about 30 Å (Fig. 1d). Presumably, the observed jump-wise lateral movement of the GB dislocation is a result the action of field-induced stress [10, 16] of about 15 GPa.

The analysis of the FIM images of the ½<110> GB dislocation in Fig. 1a–c and of other (more than 10^3^) images of non-CSL GB with a misorientation angle of *ω*_*Σ*9_/2 = 19.47*°* does not reveal breaks of spiral atomic steps, which are usually observed in the FIM images of a dissociated dislocation in the area of a stacking fault [10]. Therefore, it follows that the cores of ½<110> grain-boundary dislocations, within the limits of the resolution of the field ion microscope (2.7 Å), usually are not split. However, after the jump-like slip of the dislocation along the GB, one can see in Fig. 1e the stepped spiral characteristic of the trace of the intersection with the surface of the stacking fault related to a dissociated dislocation (shown by an arrow). The possible reason for the transformation from an undissociated to dissociated GB dislocation could be the changing of the structure of dislocation core in the GB regions with different translation states. The observed inconstancy of the splitting behavior of GB dislocations could be connected with the influence of the local GB deformation of the bicrystal under the action of field-induced ultrahigh stresses near the theoretical strength of tungsten.

The quantitative interpretation of FIM images was based on geometrical models which assume that two closely spaced spherical shells can describe those surface atoms giving rise to FIM image formation [10]. In terms of this model, the atoms protruding beyond an outer hemisphere are removed since a local electric field is greater than the threshold evaporation field. An atomic structure of GBs was analyzed by a direct consideration of their effect on the microtopography of the specimen. The most substantial effect due to GBs arises from the displacement which they produce in direction normal *n* to the specimen surface at their line of emergence.

In crystallographic lattice with unit cell vectors, *a*, *b*, and *c*, the rigid-body shift at the GB is *T* = *ua + vb + wc* where *u*, *v*, and *w* are dimensionless constants. A subatomic step formed at the line of intersection of the GB and the surface formed by field evaporation is equal to *Tn* and may be expressed in terms of a number of interplanar distances *qd*_*hkl*_, where

1 $$ q = hu+kv+lw. $$

A curved atomically smooth spherical tip of a specimen consists of the series of concentric rings arising from the atoms on the edge of crystallographic planes. The deviation from a precise round form is mainly the discrete atomic structure of the specimens. The atomic steps are composed of a set of line ledges, and observed rings are actually the polygons bounded by close-packed atomic rows. In the cases when more close-packed crystallographic planes ({110}, {100}, and {211} for bcc metals) are predominant on either side of the GB trace, the rigid-body shifts of grains along GBs can be determined using the indirect magnification phenomenon [10]. Subatomic displacements of crystallographic planes of adjacent grains in a direction normal to the surface are usually accompanied by stepwise lateral shifts of the circular atomic steps and changing of their local radii ρ.

The rigid-body shift of grains at GBs can be determined following the same methods based on the indirect magnification phenomenon as that outlined in respect of stacking faults and dissociated dislocations [10]. For small mutual shifts of grains, the normal to surface component of the rigid-body translation in a direction normal to surface formed by field evaporation, rigid translations is [17]

2 $$ Tn=\frac{\rho \varDelta \rho }{r}, $$

where Δρ is the difference between the step radii in adjacent grains and *r* is the radius of curvature of the specimen. The radius *r* was determined using the ring counting method [10]. But, this method does not take into account the anisotropy of field evaporation and gives exact results assuming a spherical envelope of the tip shape. The studied facet {211} consists of the close-packed atomic chains <111> with interatomic distance of 0.273 nm and the inter-chain distance of 0.446 nm. The number of chains on the facet is known exactly. So, we can calculate precisely the diameter (and radius ρ) without random errors.

**Fig. 2:**
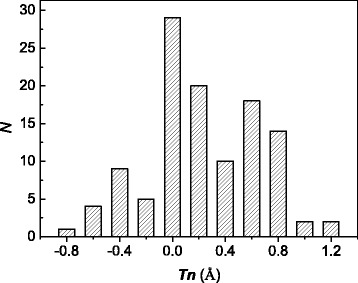
The frequency of occurrence as a function of the full vector of rigid-body shift along the GB in direction normal to the <110> misorientation axis

Experimental distribution of the rigid-body translation is presented in terms of a histogram where the frequency of the shift in a given interval is plotted as a bar graph (Fig. 2). The frequency of occurrence is shown as a function of the full vector of rigid-body shift along the GB in direction normal to the <110> direction for an asymmetric special non-CSL GB with a misorientation angle of *ω*_*Σ*9_/2. The full vectors were calculated using Eq. (2) taking into account the *Tn*_110_ = 0 condition. The comparatively broad probability distribution near the zero value of the full vector of rigid translation is evidence of the existence of multiple translation states of special non-CSL GBs.

**Fig. 3:**
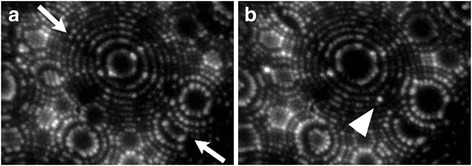
FIM images of special non-CSL GB obtained at the same image conditions as in Fig. *1* (**a**) before and (**b**) after field evaporation of monoatomic layer. The *arrow* points at the self-interstitial atom

Investigations of GBs in nonequilibrium states by field ion microscopy have the unique advantage resulting from the possibility to resolve in-plane displacements of individual atoms at imperfect interfaces. During drawing of the tungsten wire up to an elongation strain of 10^2^–10^3^ at moderate temperatures, crystal lattice dislocations and point defects interact with interface grain boundaries. The absorption of lattice dislocations by high-angle grain boundaries can be described in terms of the dissociation controlled by climb. As a result, a GB is likely to contain a nonequilibrium population of point defects. Very little is known of the way in which such individual point defects interact with the GBs at an atomic level [1,3]. Figure 3 illustrates the FIM images of the special non-CSL GB in the same specimen as Fig. 1. The FIM pattern in Fig. 3a is characteristic of a vast majority of FIM images of special non-CSL GBs. Along the GB track (shown by arrows), there are no FIM contrast effects, producing bright image spots in positions where they would not otherwise be expected to occur. An examination of about 10^4^ matching of {110} plane in FIM images of grain boundaries demonstrated that in most cases an ideal ring matching was accompanied by lack of the extra image points (i.e., an interstitial-like contrast) at the boundaries. That is clear evidence of localization of all kinds of relaxation displacements of individual atoms at plane-matching grain boundaries within the {110} planes. This kind of subatomic displacements is invisible in FIM images of the boundaries intersecting the {110} surface facets.

So, FIM study showed that {110} planes of adjoining grains are perfectly connected at the special GBs. On these special non-CSL GBs, the in-plane vectors of GB atomic relaxation have zero components along the <110> misorientation axis. Nevertheless, there are a few GB self-interstitial atoms which showed considerable relaxation in the direction perpendicular to the {110} plane. Figure 3b demonstrates a self-interstitial atom at this GB (pointed to by an arrow head). The self-interstitial atom was uncovered at the specimen surface by field evaporation of surface layers. This atom is related to neither adjoining crystal lattices. The atom is placed in the middle between two {110} atomic steps. So, in this case, the displacement in the <110> direction is equal to 1.1 Å, corresponding to one-half the {110} interplanar spacing. Analogical GB displacements of self-interstitial atoms were observed in the range of 0.3–1.1 Å.

The concentration of the GB self-interstitial atoms (SIAs) was estimated by dividing the number of the interstitial atoms by a corresponding GB area calculated in the process of controlled field evaporation. In view of the fact that the SIA segregation mainly localized in a GB monolayer [10], the fractional occupancy of SIAs can be expressed well in terms of atomic monolayers (ML). The calculated fractional occupancy of SIAs on a special non-CSL GB with a misorientation angle of *ω*_*Σ*9_/2 = 19.47 is equal to 9 × 10^−3^ ML corresponding to the GB SIA concentration of (9 ± 3) × 10^16^ m^−2^.

In calculations of intergrain interaction in the case of incommensurate GBs, related atoms are assumed to be randomly distributed with the σ_1_ and σ_2_ planar atomic density in the first and second grains, respectively. It is assumed that the GB energetic is represented by a sum of pair interactions in planar atomic layers parallel to the interface. To obtain an analytical expression for the intergrain cohesive energy, the exponential pair potential [8] was used:

3 $$ v(r)={\displaystyle \sum_p{D}_p \exp \left(-{\alpha}_pr\right)}, $$

where *r* is the interatomic distance, *D*_*p*_ is 5.169 × 10^3^ and −143.12 eV and α_*p*_ are 2.8232 and 1.4116 Å^−1^ for *p* = 1 and 2, respectively.

The number of atoms in crystallographic plane 1 interacting with a given atom in the plane 2 entering the range (*r*, *r + dr*) equals to 2*πσ*_1_*rdr.* There, *r*^2^ + *z*^2^ = *ρ*^2^ and *z* is the distance between planes 1 and 2 of adjacent grains. Using the exponential pair potential (3), the binding energy of an atom in grain 2 with all atoms in the plane of grain 1 can be expressed form as follows:

4 $$ {w}_a(r)={\sigma}_1{\displaystyle \underset{0}{\overset{2\pi }{\int }}{\displaystyle \underset{0}{\overset{\infty }{\int }}{\displaystyle \sum_p{D}_p \exp \left(-{\alpha}_pr\right)}\cdot rdrd\phi}} $$

Changing the variables of integration through Eq. (4) one obtains

5 $$ {w}_a(z)=2\pi {\sigma}_1{\displaystyle \sum_p{D}_p\left(z/{\alpha}_p+1/{\alpha}_p^2\right) \exp \left(-{\alpha}_pz\right)} $$

The energy of interaction of all atoms in both planes 1 and 2 is equal to

6 $$ {w}_{pl}(z)=2\pi {\sigma}_1{\sigma}_2{\displaystyle \sum_p{D}_p\left(z/{\alpha}_p+1/{\alpha}_p^2\right) \exp \left(-{\alpha}_pz\right)}. $$

Taking into account the interactions of all atomic layers labeled as *j* and *k* parallel to the GB surface in the both adjoining grains, the GB cohesive energy expressed in closed form is as follows

7 $$ W(r)=2\pi {\sigma}_1{\sigma}_2{\displaystyle \sum_{j,k}{\displaystyle \sum_p{D}_p\left({z}_{j,k}/{\alpha}_p+1/{\alpha}_p^2\right) \exp \left(-{\alpha}_p{z}_{j,k}\right)}}, $$

where *z*_*j,k*_ is the distance between *j* and *k* atomic layers including the grain-boundary expansion.

**Fig. 4:**
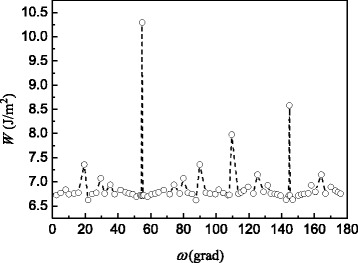
The dependence of cleavage energy on misorientation angle around the [110] axis for the {211}/{*hkl*} asymmetric tilt GBs

The cleavage energy (*W*_*c*_ = –*W*) was calculated in approximation of the rigid-body relaxation by minimizing of the *W* with respect to rigid boundary expansion. In Fig. 4, the estimated cleavage energy is represented as function of misorientation angle around the <110> axis for the asymmetrical incommensurate GBs with one grain terminated by the {211} plane. Other planes are the geometrically necessary complements to this plane, depending on the misorientation relationships. The first maximum of the cleavage energy in Fig. 4 corresponds to a special non-CSL angle *ω*_*Σ*9_/2 = 9.47*°* oriented along the $\left(1\overline{1}\overline{2}\right)/\left(1\overline{1}\overline{1}\right)$ boundary plane. It is in overall agreement with the above conclusion that the special non-CSL boundaries are not subjected to preferential field evaporation and they have a relatively a high cleavage energy.

## Conclusions

Based on the results of our study of heavily drawn ultrafine-grained tungsten with a significant population of earlier revealed a new type of special non-CSL boundaries between grains terminated by a low index plane and the medium index complements, the following conclusions can be made:

1. Field ion study revealed that {110} planes of adjacent grains are continuously connected across the interface. So, the special non-CSL GBs are the plane-matching boundaries.
2. The in-plane vectors of rigid-body translation of adjacent grains in all studied special non-CSL GBs are normal to the <110> misorientation axis.
3. Using the FIM technique of indirect magnification phenomenon, the distribution of the translation states of the special non-CSL grain boundaries in tungsten was determined. Quantitative analysis of the grain translation along the ω_Σ9_/2 special GB revealed the existence of multiple translation states of special non-CSL GBs. Mathematical modeling showed that special incommensurate GBs with one of grain oriented along the {211} plane have comparatively high cohesive energies.
4. The grain-boundary dislocations ½<110> were revealed at the line of local mismatch of {110} atomic planes of adjacent grains. The radius of the dislocation core is less than or equal to 0.6 nm.
5. FIM study showed that {110} planes of adjoining grains are perfectly connected at the surface track of special GBs. On these special non-CSL GBs, the in-plane vectors of GB atomic relaxation have zero components along the <110> misorientation axis.
